# Threaded Antibiotic-Coated Locking Nails in Osteomyelitis-Associated Long-Bone Non-Union: Short-Term Outcomes of a Prospective Cohort

**DOI:** 10.3390/healthcare14081091

**Published:** 2026-04-20

**Authors:** Akef Obeidat, Abdal Ahmad, Akhtar Hussain, Saeed Ahmad, Hidayat Ullah, Mahmood Ul Hassan, Muhammad Abrar, Sadia Qazi

**Affiliations:** 1 Department of Anatomy, College of Medicine, Alfaisal University, Riyadh 11533, Saudi Arabia; aobeidat@alfaisal.edu; 2 Department of Medicine, Peshawar Medical College, Riphah International University, Peshawar Campus, Peshawar 25160, Pakistan; abdalmomand@gmail.com; 3 Department of Orthopedics, Peshawar Medical College, Riphah International University, Peshawar Campus, Peshawar 25160, Pakistan; ahorho6@gmail.com (A.H.); ahmadsaeed_hmc@yahoo.com (S.A.); drhushah@yahoo.com (H.U.); mail2orthopedic@yahoo.com (M.U.H.); drabrar77678@gmail.com (M.A.)

**Keywords:** osteomyelitis, infected non-union, long-bone non-union, antibiotic cement-coated intramedullary nail, threaded antibiotic-coated locking nail, fracture-related infection, limb salvage, quality of life, MDR, XDR

## Abstract

**Highlights:**

**What are the main findings?**

- Single-stage threaded antibiotic-coated locking nails were associated with 96.7% short-term infection control and 90.0% radiographic union at 6 months in 30 patients with osteomyelitis-associated long-bone nonunion.
- Pain, EQ-5D-5L, and return to work all improved substantially by Month 6, and no cement debonding, implant failure, or nephrotoxicity was observed.

**What are the implications of the main findings?**

- These short-term findings suggest a pragmatic single-stage option for infected long bone non-union in resource-constrained, high-MDR/XDR settings.
- The independent contribution of the threaded core cannot be established from this single-arm study; therefore, larger comparative trials with longer follow-up are needed.

**Abstract:**

**Background**: Long-bone non-unions complicated by osteomyelitis remain a major reconstructive and healthcare challenge, particularly in resource-limited settings with a high prevalence of multidrug-resistant (MDR) pathogens. Conventional staged management is associated with a prolonged treatment burden, repeated procedures, and delayed functional recovery. This study evaluated the clinical, radiological, functional, and short-term safety outcomes of a single-stage approach using custom-threaded antibiotic-coated locking nails (TACLNs) in a high-resistance cohort. **Methods**: This prospective single-center cohort study enrolled 30 adults with osteomyelitis-associated femoral or tibial nonunion at a tertiary hospital in Peshawar, Pakistan. All patients underwent radical debridement and single-stage stabilization with a chest tube mold TACLN loaded with vancomycin and gentamicin, with culture-directed adjunctive antibiotics for resistant organisms. Outcomes were assessed at baseline, Weeks 3 and 6, and Month 6 using inflammatory markers, RUST score, VAS pain, EQ-5D-5L, ASAMI criteria, and return to work or usual activity. No formal sample size calculation was performed, and this study was exploratory in nature. **Results**: The cohort (mean age 44.9 ± 9.9 years) had a challenging microbiological profile, with 40.0% MDR and 13.3% extensively drug-resistant (XDR) infections. By Month 6, short-term infection control was achieved in 96.7% of patients, with significant reductions in ESR and CRP (both *p* < 0.001). Radiographic union was achieved in 90.0% of cases at a mean of 18.6 weeks, and the mean RUST score improved from 4.87 to 10.43 at the final follow-up. The VAS pain decreased from 5.23 at week 3 to 0.73 at month 6, EQ-5D-5L improved from 0.39 to 0.84, and 90.0% returned to work or usual activity by month 6. No cement debonding, implant failure, or nephrotoxicity was noted. **Conclusions**: In this single-arm exploratory cohort, TACLNs were associated with favorable short-term infection control, radiographic union, and functional recovery in osteomyelitis-associated long-bone nonunion, including in an MDR/XDR setting. The independent contribution of the threaded core design cannot be established. Larger multicenter comparative studies with longer follow-ups are needed to confirm the durability and implementation feasibility.

## 1. Introduction

Managing long bone non-unions complicated by concurrent osteomyelitis remains one of the most demanding scenarios in orthopedic trauma surgery. The coexistence of mechanical instability and chronic infection creates a self-perpetuating cycle that routinely defeats both standard fixation and systemic antibiotic therapy [1,2]. While nonunion is conventionally defined as the failure of fracture healing beyond nine months without radiological progression for at least three months, this threshold was developed for aseptic fracture healing and does not fully apply when active osteomyelitis is present; the coexistence of mechanical failure and ongoing infection creates an independent indication for surgical intervention regardless of time elapsed [3]. The burden of this condition falls disproportionately on working-age adults, particularly in low- and middle-income countries (LMICs), where high-energy road traffic injuries are the dominant mechanism [4]. Fracture-related infections complicate an estimated 1–2% of closed fractures and up to 30% of open fractures globally; infected non-unions represent the most refractory subset of this spectrum [5]. In resource-limited settings with a high antimicrobial resistance burden, MDR and XDR organisms account for a substantial proportion of post-traumatic osteomyelitis isolates, substantially narrowing treatment options and prolonging care [6,7,8]. Prior reports on infected long-bone nonunion and related weight-bearing long-bone infections have described encouraging outcomes with antibiotic cement-impregnated intramedullary constructs, supporting continued interest in this strategy [9,10]. In these settings, the healthcare burden extends beyond the operating theatre; prolonged disability, repeated admissions, delayed recovery, and loss of economic productivity place sustained pressure on patients, families, and already constrained health systems.

Most orthopedic infections involve biofilm-forming bacteria. Once established on implant surfaces or devitalized bone, biofilms contribute to persistent infection by limiting the effectiveness of host immune clearance and systemic antimicrobial therapy [11]. While Staphylococcus aureus, including both methicillin-susceptible (MSSA) and methicillin-resistant (MRSA) strains, is commonly isolated in post-traumatic osteomyelitis, polymicrobial infections and multidrug-resistant (MDR) profiles are frequently encountered in clinical practice [1,12]. Technical approaches for constructing antibiotic cement-coated interlocking intramedullary nails have also been described as surgeons have sought to improve local infection control and implant stability in complex cases [13]. This microbiological complexity further increases the treatment burden by narrowing the antibiotic options, prolonging care, and increasing the risk of persistent infection, repeated procedures, and treatment failure.

Traditional management has long relied on a staged paradigm: eradicating the infection first, followed by definitive fixation and bone reconstruction [14,15]. However, this approach, which typically spans several months, carries a risk of substantial morbidity. Patients often endure prolonged periods of non-weight bearing, multiple anesthetic exposures, repeated debridement, extended courses of treatment, and considerable economic productivity losses. These demands can undermine adherence, delay functional recovery, and complicate follow-up, particularly in resource-constrained environments. Consequently, a single-stage procedure that simultaneously addresses infection, instability, and dead space has been a longstanding surgical goal [16].

The antibiotic cement-coated intramedullary nail (ACCIN) concept, described by Paley and Herzenberg for intramedullary infection management, addresses these requirements [15,17]. The implant simultaneously stabilizes the fracture and fills the dead space, aligning with the key Cierny–Mader treatment principles in a single operative session [18]. In addition, antibiotic-impregnated polymethyl methacrylate (PMMA) coating can provide high local antibiotic delivery while maintaining mechanical support [19]. Favorable outcomes with antibiotic-impregnated cement-coated intramedullary nails have also been reported in chronic osteomyelitis, further supporting the broader applicability of this strategy [20]. From a healthcare perspective, this combined approach is attractive because it may reduce procedural fragmentation, support earlier mobilization, and lessen the cumulative burden associated with prolonged staged care.

Surgeons have described multiple fabrication techniques that utilize guide wires, chest tubes, endotracheal tubes, bronchoscopy tubing, and silicone molds [13,14,15,16,17]. Similar constructs have been described for complex infected hindfoot reconstruction, demonstrating that cement-impregnated nail-based strategies are applicable across a range of difficult infection settings [21]. However, the choice of the inner core is highly consequential in this regard. Nails fabricated around smooth guidewires demonstrated alarming cement debonding rates, as high as 47% at removal. In contrast, constructs built on threaded rods or standard interlocking nails showed 0% debonding under identical conditions [22]. Cho et al. demonstrated the efficacy of antibiotic-coated hinged threaded rods in a 27-patient cohort, reporting zero cement debonding events and an 88.9% negative culture conversion rate, confirming the mechanical advantage of the threaded interface [23]. In practical terms, implant integrity from reduced debonding directly affects procedural reliability, ease of removal, and risk of avoidable re-intervention.

Despite the growing body of evidence, comparative data remain limited. A 2022 meta-analysis found no statistically significant difference in union rates or infection eradication between ACCIN and conventional comparator interventions, although the authors emphasized the paucity of well-designed trials [3]. More recent series have also reported favorable infection eradication and union outcomes in fracture-related infection, nonunion, infected bone defects, and infected tibial nonunion treated with antibiotic cement-coated nails [24,25,26]. Published studies consistently report infection control rates of 70–96% and union rates of 70–90% across infected long bone non-union and related osteomyelitis settings [1,2,3,4,9,10]. Recent literature from Pakistan has begun to highlight the utility of this approach in regional contexts [27]. While commercially available antibiotic-coated nails offer standardized formulations and shorter operating times, intraoperatively fabricated custom nails allow for antibiogram-directed antibiotic selection, which is a material advantage when multidrug-resistant (MDR) and extensively drug-resistant (XDR) organisms predominate [28]. This flexibility may be particularly important in settings where resistance patterns are heterogeneous, and access to proprietary implants is limited.

The technique evaluated in this study utilizes a custom threaded antibiotic-coated locking nail (TACLN) fabricated intraoperatively using a chest tube mold. Unlike conventional ACCIN constructs built around smooth guidewires, the TACLN uses a threaded inner rod to maximize the mechanical interlock between the cement mantle and nail substrate, directly addressing the debonding failure mode that limits smooth-core constructs [22,23]. Our baseline antibiotic formulation (2 g vancomycin and 240 mg gentamicin) combined with culture-directed adjuncts for resistant organisms provides broad-spectrum coverage across susceptible, MDR, and XDR profiles. This study prospectively evaluated the short-term clinical, radiological, functional, and safety outcomes of single-stage treatment with an intraoperatively fabricated TACLN in 30 patients with osteomyelitis-associated long bone nonunion at a tertiary hospital in Peshawar, Pakistan.

## 2. Materials and Methods

### 2.1. Study Design and Setting

This prospective, single-center cohort study was conducted at Mercy Teaching Hospital, Peshawar, Pakistan, between August 2025 and February 2026. The study evaluated the threaded, antibiotic-coated locking nail intervention under routine tertiary care conditions in a resource-constrained setting, with an emphasis on infection control, patient recovery, radiological healing, and short-term safety. Reporting followed the STROBE principles [29], and the completed STROBE checklist is provided as Supplementary Table S1. No formal sample size calculations were performed. This study was exploratory in nature, and all findings should be interpreted as preliminary pending confirmation in larger controlled studies. Ethical approval was granted by the Prime Foundation Pakistan Institutional Review Board on 2 August 2025 (PRIME/IRB/2025-1175), and all participants provided written informed consent.

### 2.2. Participants and Eligibility Criteria

Adult patients (≥18 years) presenting with long-bone nonunion (femur or tibia) between August and September 2025 were considered for inclusion. In this study, nonunion was defined as radiologically confirmed failure of progressive fracture healing in the presence of microbiologically confirmed active osteomyelitis requiring surgical intervention. Consistent with the clinical rationale described in the Introduction, the conventional 9-month time-based threshold was not applied as the primary eligibility criterion; the coexistence of mechanical failure and active infection was treated as an independent surgical indication regardless of time elapsed since the index injury.

Osteomyelitis was confirmed microbiologically using intraoperative deep-tissue culture. Anatomical involvement was staged using the Cierny–Mader classification [18]. Multidrug resistance (MDR) was defined as acquired non-susceptibility to at least one agent in three or more antimicrobial categories, and extensive drug resistance (XDR) was defined as non-susceptibility to at least one agent in all but two or fewer categories, in accordance with international consensus criteria [30].

The exclusion criteria were age < 18 years, pathological fracture, active malignancy, immunosuppressive therapy, and incomplete follow-up data. No patients were excluded after enrolment; all 30 patients who met the eligibility criteria and consented were included in the final analysis.

### 2.3. Surgical Intervention

All procedures were performed under general anesthesia by a single senior surgeon. The operative sequence comprised radical debridement, intramedullary reaming, and single-stage implantation of a custom TACLN construct. Intramedullary reaming was performed sequentially to 1 mm above the isthmus diameter, as determined by the largest reamer passing the isthmus with tactile cortical resistance. The threaded inner rod was sized to match the reamed canal, ranging from 10 to 12 mm for femoral cases and 8 to 10 mm for tibial cases. The chest tube mold was sized to achieve a cement mantle of approximately 2–3 mm circumferentially. The construct was secured in a static locking configuration using proximal and distal interlocking screws. For additional construct stability, threaded nuts were applied to the rod ends, maintaining axial alignment and rotational stability. The fabrication sequence, from the bare threaded rod and antibiotic-loaded PMMA components to the completed construct, is illustrated in Supplementary Figure S1. Segmental bone defects of less than 2 cm were managed by acute shortening with end-to-end bony apposition where limb-length considerations permitted. Defects measuring 2 to 3 cm were supplemented with iliac crest bone graft, whereas defects greater than 3 cm were reconstructed using femoral head allograft. An antibiotic-laden cement mantle was used to fill and sterilize any residual dead space. Soft-tissue closure was achieved by primary layered closure in wounds with adequate tissue quality and absence of tension. Wounds with significant contamination, periosteal devascularization, or soft-tissue deficit were managed with negative-pressure wound therapy at 125 mmHg continuous pressure pending delayed primary closure.

The PMMA formulation consisted of 2 g vancomycin and 240 mg gentamicin per 40 g of PMMA as the standard baseline load [31]. Where intraoperative cultures or prior microbiology indicated MDR or XDR organisms, the antibiotic selection was adjusted according to the susceptibility profile and the thermal stability of the agent (e.g., meropenem or fosfomycin were added for carbapenem-resistant Gram-negative isolates; no additional agents were added for organisms susceptible to the baseline combination). Postoperative systemic antibiotics were administered intravenously for a minimum of 4 weeks, followed by an oral consolidation phase for a further 2–4 weeks, with the total duration individualized to the intraoperative culture result and the recommendation of the treating infectious disease team; the mean total antibiotic duration across the cohort was 7.1 ± 2.5 weeks. Partial weight-bearing with crutch support was permitted at a median of week 4 postoperatively, progressing to full weight-bearing based on clinical and radiological assessments, achieved at a mean of 25.9 ± 1.3 weeks. Formal physiotherapy commenced within the first 48 h postoperatively, initially comprising passive range-of-motion exercises and non-operative limb conditioning, with progressive lower-limb rehabilitation and gait training introduced in parallel with weight-bearing advancement.

### 2.4. Variables and Data Collection

Data were collected prospectively using a standardized proforma. Baseline demographic and clinical characteristics included age, sex, affected bone, duration since last surgery, duration of nonunion, prior surgical procedures, and comorbidity burden, quantified using the Charlson Comorbidity Index (CCI) [32]. The microbiological data comprised intraoperative culture results, organism identity, and resistance classification. Bone defect size, preoperative soft-tissue condition, coronal and sagittal alignment, and open fracture grade (Gustilo-Anderson) were not systematically recorded; this limits independent assessment of baseline severity and is acknowledged as a study limitation.

Several measures were taken to reduce the potential bias in data collection and outcome assessment. Radiographs were assessed independently by two authors blinded to the clinical outcomes, with discordant assessments resolved by consensus to minimize detection bias in the primary radiological endpoint. All outcome data were collected prospectively using a standardized proforma administered at prespecified time points, reducing the risk of recall and ascertainment bias inherent to retrospective designs. The treating surgeon was not involved in the radiological or functional outcome scoring. Patient-reported outcomes (VAS pain, EQ-5D-5L, return to work) were recorded by a research team member who was independent of the surgical team. Selection bias was mitigated by the consecutive enrolment of all eligible patients presenting during the study period; no patients were excluded after enrolment. Residual confounding from unmeasured variables, including smoking status, soft tissue condition, and open fracture severity, could not be fully controlled in this single-arm observational design and is acknowledged as a limitation.

### 2.5. Outcome Measures

#### 2.5.1. Primary Outcome

The primary outcome was short-term infection control at six months, defined as the absence of clinical, laboratory, and radiological signs of active infection at the final follow-up visit.

#### 2.5.2. Radiographic Outcomes

Radiographic union was evaluated using plain anteroposterior and lateral radiographs obtained at 3 and 6 weeks and 6 months postoperatively. Union was defined as cortical bridging in ≥3 of the 4 cortices on orthogonal views, corresponding to a RUST score of ≥10 [33]. At this center, postoperative radiological review was performed at weeks 3 and 6 and at month 6; persistent absence of bridging callus across all cortices on two consecutive reviews at week 6 and month 6 was used to classify a case as radiological non-union at the final assessment. Radiographs were assessed independently by two authors, and discordant assessments were resolved by consensus. The assessors were blinded to the clinical outcomes. Healing was quantified using the Radiographic Union Score for Tibial fractures (RUST). The RUST score was applied to both tibial and femoral fractures in this study. It should be noted that the RUST score was originally validated for tibial fractures only; its application to the 20 femoral cases in this cohort (66.7%) represents off-label use and constitutes a methodological limitation of this study. Scores in femoral cases should be interpreted with caution, and the use of a femur-specific or generic long-bone union scale in future studies is recommended. Limb-length discrepancy (LLD) was assessed clinically by direct tape measurement from the anterior superior iliac spine to the medial malleolus with the patient in the supine position, confirmed by the block test in the standing position where feasible. Both angular deformity and LLD were used to derive the deformity and LLD subscores of the ASAMI bone results.

#### 2.5.3. Functional and Patient-Reported Outcomes

Functional outcomes were assessed using the ASAMI scoring system, the visual analog scale (VAS) for pain, and the EQ-5D-5L health utility index [34]. The VAS for pain was recorded preoperatively (baseline) and at each follow-up visit (weeks 3, 6, and month 6). Where preoperative VAS was not available, the week 3 measurement was used as the comparator baseline; this is acknowledged as a limitation and may underestimate the true pain reduction achieved. Return to work or usual activity was recorded as a binary outcome at weeks 3 and 6 and at month 6. For this study, return to work was defined as resumption of the patient’s pre-injury occupational or usual daily activity at a level functionally equivalent to their pre-injury status. Patients who had not resumed any occupational or usual activity by month 6 were classified as not returned, regardless of reason.

### 2.6. Statistical Analysis

Data were analyzed using IBM SPSS Statistics for Windows, Version 28.0 (IBM Corp., Armonk, NY, USA). Continuous variables are presented as mean ± standard deviation (SD) or median with interquartile range (IQR) according to the distribution normality assessed by the Shapiro–Wilk test. Categorical variables are presented as frequencies and percentages.

For continuous outcomes measured at three or more time points (ESR, CRP, and RUST scores at Baseline, Week 3, Week 6, and Month 6; VAS pain score at Week 3, Week 6, and Month 6), longitudinal change was evaluated using a one-way repeated-measures analysis of variance (RM-ANOVA). Mauchly’s test of sphericity was applied; where sphericity was violated (*p* < 0.05), the Greenhouse–Geisser correction was applied to degrees of freedom and *p*-values. Statistically significant RM-ANOVA results were followed by pairwise post hoc comparisons with Bonferroni correction. Effect sizes were reported as partial eta-squared (η^2^g). For EQ-5D-5L, which was measured at two timepoints only (Baseline and Month 6), change was evaluated using a paired *t*-test with Cohen’s *dz* as the effect size measure [35]. For binary repeated outcomes (return to work or usual activity), changes across time points were evaluated using McNemar’s exact test. Statistical significance was set at *p* < 0.05. No formal sample size calculation was performed; this study was exploratory, and all inferential results should be interpreted accordingly.

## 3. Results

**Figure 1:**
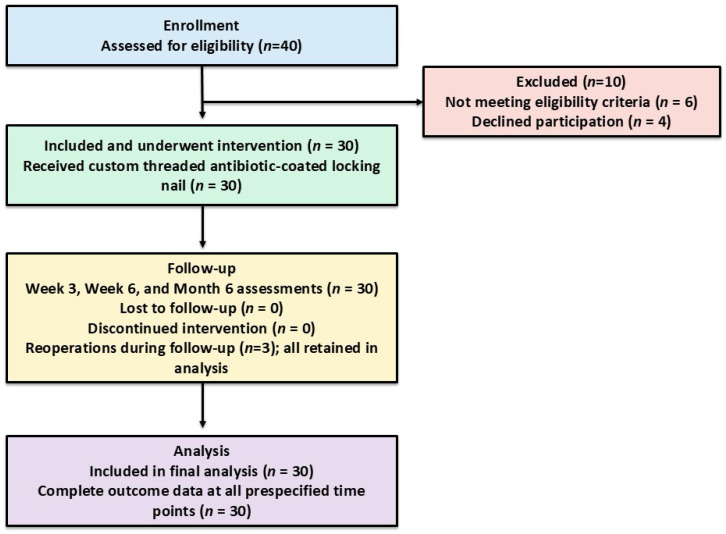
STROBE flow diagram of patient screening, allocation, follow-up and analysis.

This prospective single-center cohort study screened 40 patients, of whom 30 met the eligibility criteria, underwent intervention, and were included in the final analysis (Figure 1).

All 30 patients had complete data for each variable at the prespecified measurement timepoints, with no loss to follow-up or missing data in the planned analysis. Primary inferential contrasts were limited to baseline versus month 6 for biological and radiological variables and week 3 versus month 6 for functional variables.

### 3.1. Baseline Patient and Infection Characteristics

The baseline demographic, clinical, and microbiological characteristics are summarized in Table 1. The cohort included 18 men (60.0%) and 12 women (40.0%), with a mean age of 44.9 ± 9.9 years and a mean BMI of 23.1 ± 2.9 kg/m^2^. Femoral nonunion accounted for 66.7% of cases, whereas tibial nonunion accounted for 33.3%. The median Charlson Comorbidity Index was 0 (IQR 0–1), and the mean duration since the last surgery was 3.0 ± 1.4 months.

The microbiological profile was notable for resistant organisms. Fourteen infections (46.7%) were classified as susceptible, 12 (40.0%) as multidrug-resistant (MDR), and 4 (13.3%) as extensively drug-resistant (XDR). The most frequently identified pathogen groups were polymicrobial or other organisms (70.0%), followed by methicillin-susceptible Staphylococcus aureus (16.7%) and methicillin-resistant Staphylococcus aureus (13.3%).

### 3.2. Infection Control and Systemic Response

**Figure 2:**
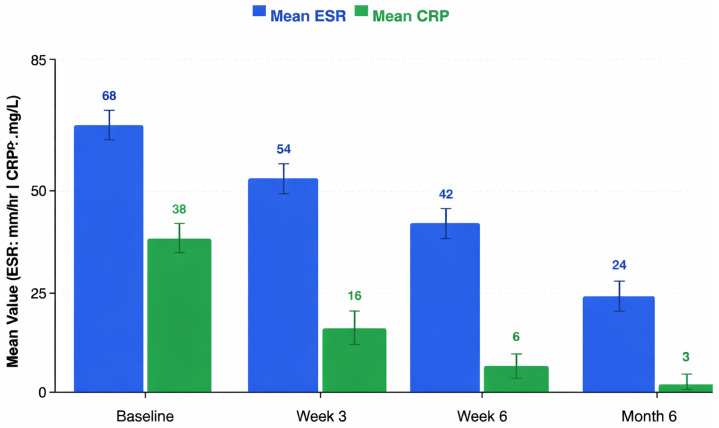
Progression of systemic inflammatory markers. A grouped bar graph showing mean erythrocyte sedimentation rate (ESR, mm/h) and C-reactive protein (CRP, mg/L) at baseline, Week 3, Week 6, and Month 6. Error bars represent 95% confidence intervals. Both markers showed substantial longitudinal declines over the follow-up period. Cohen’s *dz* effect sizes for the baseline-to-month-6 change were 4.21 for ESR and 3.18 for CRP.

Following intervention with the custom-threaded antibiotic-coated locking nail, systemic markers of infection and inflammation decreased markedly over time (Table 2; Figure 2).

#### 3.2.1. Inflammatory Markers

The mean baseline ESR was 67.80 ± 9.73 mm/h, which decreased to 54.40 ± 10.12 mm/h at week 3, 41.70 ± 11.27 mm/h at week 6, and 24.40 ± 11.59 mm/h at month 6. The mean baseline CRP decreased from 39.27 ± 11.26 mg/L to 16.80 ± 3.75 mg/L at week 3, 5.77 ± 4.57 mg/L at week 6, and 3.47 ± 3.41 mg/L at month 6.

For ESR, Mauchly’s test indicated a violation of sphericity (W = 0.239, *p* = 1.82 × 10^−7^); after Greenhouse–Geisser correction (ε = 0.568), the RM-ANOVA was highly significant (F = 336.86, *p*-GG = 3.39 × 10^−28^, η^2^g = 0.690). Bonferroni-corrected pairwise comparisons confirmed that every timepoint differed significantly from every other (all *p*-corr < 2.82 × 10^−15^).

For CRP, sphericity was also violated (W = 0.067, *p* = 1.11 × 10^−14^); after Greenhouse–Geisser correction (ε = 0.415), the RM-ANOVA was significant (F = 217.91, *p*-GG = 3.79 × 10^−18^, η^2^g = 0.827). All pairwise comparisons were significant after Bonferroni correction (all *p*-corr ≤ 0.012), including the comparison between Week 6 and Month 6 (*p*-corr = 0.012), indicating that CRP levels continued to decline even in the final phase of follow-up.

#### 3.2.2. Serum Creatinine

Serial serum creatinine measurements remained stable throughout the follow-up period (baseline: 0.97 mg/dL; week 3: 1.07 mg/dL; week 6: 0.98 mg/dL; month 6: 0.95 mg/dL), with no clinically significant changes across any time point.

Seventeen of the 30 patients (56.7%) experienced at least one postoperative event during follow-up. The most common complications were recurrent or persistent local infection (*n* = 5, 16.7%), pain (*n* = 5, 16.7%), and wound swelling (*n* = 5, 16.7%), all of which were managed nonoperatively. Two patients (6.7%) developed postoperative septicemia, both of whom subsequently required reoperation. Three patients (10.0%) required reoperation, two with septicemia and one with a wound complication that did not resolve with conservative management. All three patients had not achieved radiographic union by month 6. Wound complications in this cohort were consistent with the expected risk profile of this patient population, in which pre-existing soft-tissue compromise, periosteal devascularization from chronic infection, and prior surgical scarring predispose patients to wound healing difficulties. No implant failures, cement debonding events, or antibiotic-related nephrotoxicity were observed.

#### 3.2.3. Postoperative Management

All patients received culture-directed systemic antibiotics administered in two sequential phases: an intravenous phase commenced immediately postoperatively and an oral consolidation phase. The mean total antibiotic duration was 7.1 ± 2.5 weeks (range, 3–11 weeks). The choice of agent was individualized according to the microbiological resistance profile. The regimens included cephalosporins, meropenem-based combinations, vancomycin with oral linezolid, and fluoroquinolone-based oral phases for susceptible Gram-negative isolates.

The median hospital length of stay was 2 days (IQR 1–2); 10 patients (33.3%) were discharged at 1 d and 20 (66.7%) at 2 days. Partial weight bearing commenced at a median of week 4 (IQR 4–5). Full weight bearing was achieved at a mean of 25.9 ± 1.3 weeks (range, 24–29 weeks).

### 3.3. Functional Recovery and Quality of Life

#### 3.3.1. Pain

The mean VAS pain score was 5.23 ± 1.22 at week 3, decreasing to 2.43 ± 1.04 at week 6 and 0.73 ± 1.11 at month 6. Sphericity was not violated (W = 0.919, *p* = 0.306). RM-ANOVA across Weeks 3 and 6 and Month 6 showed a significant effect of time (F(2,58) = 161.78, *p* = 1.88 × 10^−24^, η^2^g = 0.737). All three pairwise comparisons were significant after Bonferroni correction: week 3 vs. week 6 (*p*-corr = 1.56 × 10^−10^), week 6 vs. month 6 (*p*-corr = 3.11 × 10^−8^), and week 3 vs. month 6 (*p*-corr = 2.78 × 10^−16^), indicating a sustained and progressive pain reduction throughout the follow-up.

#### 3.3.2. Health-Related Quality of Life

Mean EQ-5D-5L increased from 0.39 ± 0.13 at baseline to 0.84 ± 0.20 at Month 6 (mean change 0.44, 95% CI 0.38 to 0.51; t(29) = 13.64, *p* = 3.82 × 10^−14^, Cohen’s *dz* = 2.49). As EQ-5D-5L was measured at only two timepoints, a paired *t*-test was used.

#### 3.3.3. Return to Work or Usual Activity

No patient had returned to work or usual activity by week 3 (0/30). This increased to 53.3% (16/30; 95% CI 34.3% to 71.7%) at Week 6 and to 90.0% (27/30; 95% CI 73.5% to 97.9%) at Month 6. The paired increase from week 3 to month 6 was statistically significant (McNemar’s exact test, *p* = 1.49 × 10^−8^). The three patients who had not returned by month 6 were the same patients who did not achieve radiographic union and required reoperation.

#### 3.3.4. ASAMI Functional Outcome

At Month 6, 66.7% of patients (20/30; 95% CI 47.2% to 82.7%) had an Excellent ASAMI functional result, while 23.3% (7/30; 95% CI 9.9% to 42.3%) had a good result. No patients were classified as Fair, and 10.0% (3/30; 95% CI 2.1% to 26.5%) were classified as Poor.

### 3.4. Radiological Healing and ASAMI Bone Outcomes

#### 3.4.1. RUST Score Progression

The mean baseline RUST score was 4.87 ± 0.78, increasing to 7.13 ± 1.25 at week 3, 9.00 ± 1.17 at week 6, and 10.43 ± 1.33 at month 6. Sphericity was not violated (W = 0.884, *p* = 0.635). RM-ANOVA showed a significant effect of time (F(3,87) = 204.52, *p* = 2.75 × 10^−39^, η^2^g = 0.794). All pairwise comparisons were significant after Bonferroni correction (all *p*-corr < 5.10 × 10^−13^), confirming progressive cortical bridging at each successive assessment.

#### 3.4.2. Union and ASAMI Bone Outcome

**Figure 3:**
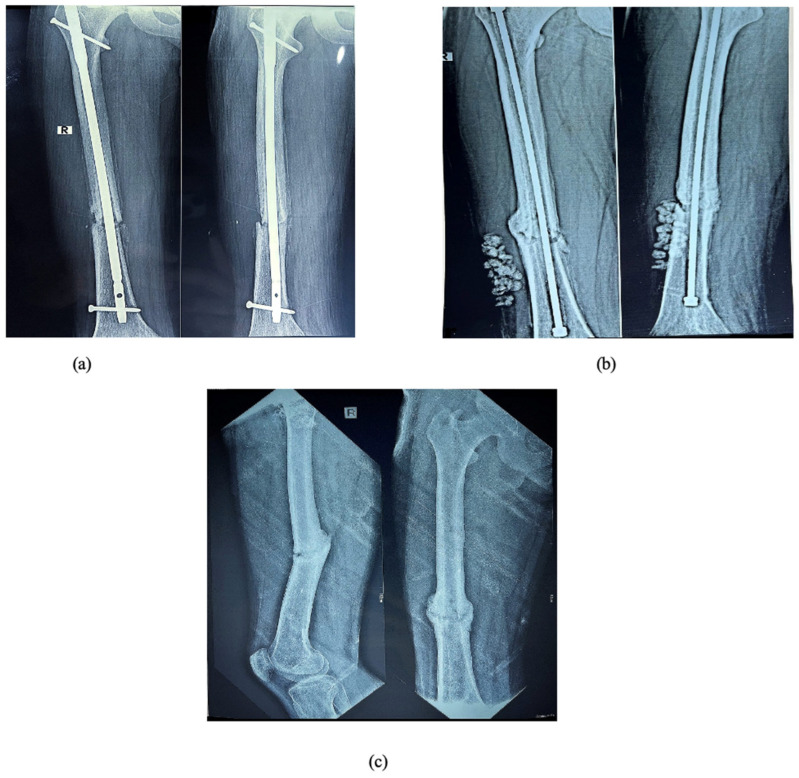
Representative serial radiographs of infected nonunion managed with a custom antibiotic-coated locking nail. (**a**) Preoperative radiograph showing infected nonunion; (**b**) third-week postoperative AP and lateral views showing the implant in situ; (**c**) six-month AP and lateral views showing progressive cortical bridging and radiographic union.

The mean time to confirm radiographic union was 18.6 weeks (SD, 2.7). By Month 6, radiographic union was achieved in 90.0% of patients (27/30; 95% CI 73.5% to 97.9%), and short-term infection control was documented in 96.7% (29/30; 95% CI 82.8% to 99.9%). Representative serial radiographs illustrating preoperative infected nonunion, postoperative implant position, and six-month radiographic union are shown in Figure 3. At Month 6, the mean residual deformity was 1.8° ± 1.7° (range 0–7°), and the limb-length discrepancy was less than 2 cm in 27/30 patients (90.0%). According to the full ASAMI bone criteria, which incorporate union status, infection control, residual deformity, and limb-length discrepancy, 90.0% of patients (27/30; 95% CI 73.5% to 97.9%) were classified as Excellent at Month 6. The remaining three patients (10.0%) were classified as poor, each meeting the criterion of non-union at the final assessment timepoint, regardless of other outcome domains.

### 3.5. Exploratory Descriptive Stratification

Formal subgroup analyses were not performed given the sample size and high overall success rates. Stratified descriptive data are presented for clinical context only and should be interpreted as hypothesis generating. See Table 3 and Table 4.

## 4. Discussion

The management of infected long bone non-unions requires simultaneous infection control, durable mechanical stabilization, and restoration of function. In this prospective cohort, single-stage treatment with custom-threaded antibiotic-coated locking nails was associated with 96.7% short-term infection control, 90.0% radiographic union, substantial pain reduction, improved EQ-5D-5L scores, and 90.0% return to work or usual activity by month 6. These results suggest that the TACLN approach supported infection control, structural healing, and functional recovery across a clinically demanding MDR/XDR cohort.

### 4.1. Infection Control in a High-Resistance Setting

The 96.7% infection control rate at Month 6 lies within the upper range reported in prior studies. Conway et al. reported that 93.8% of 96 patients treated with ACCINs for fracture-related infection achieved healed, uninfected bone [24]. Similarly, Garabano et al. reported an overall infection control rate of 90.6% in 96 patients with chronic post-traumatic osteomyelitis [28].

The marked decline in CRP, from 39.27 mg/L at baseline to 3.47 mg/L by month 6, is consistent with effective early and sustained local antibiotic delivery. This pattern is biologically plausible because PMMA-based local delivery can achieve high antibiotic concentrations at the infection site, including levels sufficient to exceed the minimum inhibitory concentration in biofilm-associated environments [31].

The microbiological profile of this cohort, including 40.0% MDR and 13.3% XDR infections, reflects a demanding clinical context particularly relevant in resource-constrained settings. A practical advantage of custom intraoperative nail fabrication is the ability to tailor the cement formulation to microbiological findings, including culture-directed adjunctive antibiotics, where appropriate [28]. In the present cohort, all patients received culture-directed systemic antibiotics across two sequential phases, with a mean total duration of 7.1 ± 2.5 weeks. The regimens were individualized according to the resistance profile and included meropenem-based combinations, vancomycin with oral linezolid, and fluoroquinolone-based consolidation phases for susceptible Gram-negative bacterial isolates. Similarly, McNally et al. reported that resistant organisms do not necessarily confer a higher recurrence risk when debridement is adequate and local antibiotic delivery is appropriately targeted [36]. The ability to combine aggressive debridement, local culture-directed antibiotic delivery, and stabilization in a single operative strategy may reduce treatment fragmentation and support more efficient care continuity in settings where access to repeated staged procedures is limited.

### 4.2. Radiological Outcomes and Use of the RUST Score

The mean RUST score increased significantly from 4.87 at baseline to 10.43 at month 6, and 90.0% of patients achieved radiographic union by 6 months. The mean time to union was 18.6 weeks, which is consistent with prior reports. Sen et al. reported union in 90.9% of cases over 3.3 to 6 months [9], while Wang et al. reported union in 95% of patients at a mean follow-up of 28.3 weeks [25].

Although the RUST score was originally developed for tibial fractures, its use in femoral non-unions in this cohort provided a practical continuous measure of cortical bridging, enabling serial assessment of healing progression beyond a simple binary union endpoint [33]. However, its application to femoral reconstruction in this study should be interpreted as pragmatic rather than formally validated.

### 4.3. Mechanical Implications of Threaded Cores

A recognized technical concern with custom-made ACCINs is cement debonding during insertion or removal. Bray et al. reported debonding in 47% of guidewire-based constructs compared with 0% in interlocking nail or threaded rod constructs [22]. Cho et al. also reported no debonding events in a 27-patient cohort using threaded rods [23].

No cement debonding was observed in this cohort, a finding consistent with both prior reports and the mechanical rationale for threaded inner cores. The threaded interface increases surface contact area and mechanical interlock between the cement mantle and nail substrate, reducing the shear forces responsible for debonding in smooth-core constructs [22,23]. The specific contribution of the threaded design cannot be isolated without a comparator group, but the zero debonding rate observed here is mechanically coherent with the threaded interface hypothesis.

### 4.4. Functional Recovery, Patient-Centered Outcomes, and Systemic Safety

Limb reconstruction should be judged by recovery of function and return to daily life, not radiographic union alone. In this cohort, EQ-5D-5L scores improved significantly, pain decreased substantially, and 90.0% of patients had returned to work or usual activity by month 6. Pain relief, restoration of mobility, and return to vocational or usual social roles are outcomes that matter directly to patients and their families, particularly in working-age populations where prolonged disability carries major personal, social, and economic consequences [37,38].

At Month 6, 66.7% of patients had Excellent ASAMI functional results, whereas 10.0% were graded as poor. These three patients also accounted for the reoperations recorded in this cohort: two developed postoperative septicemia requiring surgical intervention, and one had a wound complication that did not resolve with conservative management. None of the three patients achieved radiographic union by month 6, and their poor functional classification reflects the convergence of persistent nonunion, infectious complications, and operative reintervention. All three had MDR infections at baseline, which is clinically noteworthy even if the sample is too small to support formal comparison. Given the baseline complexity of this cohort, including resistant organisms and prior surgical burden, these functional findings remain clinically meaningful, although residual disability persisted in a subset of patients.

17 of 30 patients (56.7%) experienced at least one postoperative event during the follow-up. The most frequent complications were local infection recurrence, pain, and wound swelling, each occurring in five patients (16.7%) and managed nonoperatively. The overall reoperation rate was 10.0% (3/30). Despite the high-dose local vancomycin strategy, serial serum creatinine values remained stable during follow-up. Prior studies support the local elution capacity of antibiotic-loaded PMMA, although systemic exposure depends on the clinical context [39].

### 4.5. Healthcare and Implementation Relevance

The short-term care delivery data are worth noting. In this cohort, the median hospital length of stay was 2 days (IQR 1–2), partial weight bearing commenced at a median of week 4, and full weight bearing was achieved at a mean of 25.9 ± 1.3 weeks. These figures compare favorably with the prolonged immobilization and repeated admissions typically associated with staged management [40]. Although this study did not directly measure costs or formal health economic outcomes, a single-stage strategy that delivers debridement, stabilization, and local antibiotic delivery simultaneously may reduce procedural fragmentation and cumulative treatment burden. These outcomes warrant formal evaluation in future comparative studies.

### 4.6. Strengths and Limitations

This study had several strengths. It was conducted prospectively under routine tertiary care conditions and evaluated a clinically difficult cohort with substantial MDR/XDR burden, reflecting real-world practice in a resource-constrained environment. In addition to infection control and radiographic union, the study incorporated patient-reported quality of life, pain, return to work or usual activity, and short-term renal safety, providing a broader outcome profile than structural healing alone.

This study has several limitations. First, the sample size was small and derived from a single center, which limits its generalizability. Second, the 6-month follow-up period was sufficient to assess early union and short-term infection control but not late recurrence; prior work has shown that recurrence may occur well beyond this period [36]. Accordingly, the infection findings reported here are best interpreted as short-term control rather than definitive, long-term eradication. Third, the absence of a control group limits causal inference, including the assessment of the independent effect of the threaded core design relative to debridement, stabilization, and antibiotic delivery. Fourth, although RUST scoring is useful for serial assessment, its application to femoral fractures remains outside its original validation context. Fifth, smoking status, open fracture classification, and formal soft-tissue staging were not systematically recorded; the absence of these variables limits risk stratification and may affect the interpretability of wound complications and union rates. Full-length standing radiographs were not available at this center; therefore, limb-length discrepancy was assessed by clinical tape measurement, which is less precise than scanography and may have introduced a small measurement error in the ASAMI bone sub-scores. Sixth, the sample size did not permit a formal subgroup analysis by fracture site or resistance profile. Descriptively, all three cases of nonunion, the single infection failure, and all three poor functional outcomes occurred in patients with MDR infections; this pattern is clinically noteworthy but cannot be tested reliably in a cohort of 30 patients and warrants prospective evaluation in larger studies. Finally, although the discussion highlights the potential care delivery and resource advantages of a single-stage approach, this study did not directly evaluate the hospital length of stay, cost, reoperation burden, or formal health economic outcomes.

## 5. Conclusions

In this single-arm exploratory cohort of 30 patients with osteomyelitis-associated long-bone nonunion, single-stage treatment with custom-threaded antibiotic-coated locking nails was associated with favorable short-term infection control, radiographic union, pain reduction, improved quality of life, and return to work or usual activity by month 6, including in patients with MDR and XDR infections. No cement debonding, implant failure, or antibiotic-related nephrotoxicity was observed. These findings are limited by the single-arm design, six-month follow-up, and single-center setting; the independent contribution of the threaded core design could not be established. Larger multicenter comparative studies with longer follow-up are needed to confirm durability, implementation feasibility, and cost-effectiveness, and to determine the specific mechanical contribution of the threaded core relative to debridement, stabilization, and antibiotic delivery.

## Figures and Tables

**Table 1:** Baseline Patient Demographic and Clinical Characteristics (*N* = 30).

Characteristic	Cohort (*N* = 30)
Age (years), Mean ± SD	44.9 ± 9.9
Gender, *n* (%)	
Male	18 (60.0%)
Female	12 (40.0%)
Body Mass Index (BMI), Mean ± SD	23.1 ± 2.9
Charlson Comorbidity Index (CCI), Median (IQR)	0 (0–1)
Involved Bone, *n* (%)	
Tibia	10 (33.3%)
Femur	20 (66.7%)
Duration since last surgery (months), Mean ± SD	3.0 ± 1.4
Bone Defect Size (cm), Mean ± SD	3.21 ± 0.56
Prior Treatment, *n* (%)	
Intramedullary nailing (IMN)	16 (53.3%)
External fixation	13 (43.3%)
Debridement + IMN	1 (3.3%)
Comorbidities, *n* (%)	
None	17 (56.7%)
Diabetes mellitus	7 (23.3%)
Hypertension	6 (20.0%)
Obesity (BMI ≥ 30)	4 (13.3%)
Number of Prior Surgeries, Median (Range)	1 (1–2)
Primary Pathogen Profile, *n* (%)	
Methicillin-resistant *S. aureus* (MRSA)	4 (13.3%)
Methicillin-susceptible *S. aureus* (MSSA)	5 (16.7%)
Polymicrobial/Other *	21 (70.0%)
Pathogen Resistance Profile, *n* (%)	
Susceptible	14 (46.7%)
Multidrug-Resistant (MDR)	12 (40.0%)
Extensively Drug-Resistant (XDR)	4 (13.3%)

Note: Continuous variables are presented as mean ± SD or median (IQR), as appropriate. Categorical variables are presented as numbers (*n*) and percentages (%). Polymicrobial/other * infections included *Klebsiella pneumoniae* (*n* = 7, 23.3%), *Pseudomonas aeruginosa (n* = 6, 20.0%), *Escherichia coli* (*n* = 6, 20.0%), and mixed infections (*n* = 2, 6.7%). Smoking status, open fracture classification, and formal soft-tissue staging were not systematically recorded and are acknowledged as study limitations.

**Table 2:** Longitudinal Progression of Clinical, Radiological, and Functional Outcomes (*N* = 30).

Parameter	Initial Assessment (Mean ± SD)	Month 6 Assessment (Mean ± SD)	Mean Change ± SD (95% CI of Change)	Exact *p*-Value	Effect Size (Cohen’s *dz*, 95% CI)
ESR (mm/h)	67.80 ± 9.73	24.40 ± 11.59	−43.40 ± 10.31 (−47.25 to −39.55)	3.38 × 10^−20^	4.21 (3.09 to 5.33)
CRP (mg/L)	39.27 ± 11.26	3.47 ± 3.41	−35.80 ± 11.24 (−40.00 to −31.60)	6.50 × 10^−17^	3.18 (2.30 to 4.07)
RUST Score	4.87 ± 0.78	10.43 ± 1.33	5.57 ± 1.38 (5.05 to 6.08)	1.12 × 10^−19^	4.03 (2.95 to 5.11)
EQ-5D-5L Score	0.39 ± 0.13	0.84 ± 0.20	0.44 ± 0.18 (0.38 to 0.51)	3.82 × 10^−14^	2.49 (1.77 to 3.21)
VAS Pain Score *	5.23 ± 1.22	0.73 ± 1.11	−4.50 ± 1.43 (−5.03 to −3.97)	9.26 × 10^−17^	3.14 (2.27 to 4.01)

Note: Effect sizes in this table represent Cohen’s *dz* for the initial assessment-to-Month 6 comparison; partial eta-squared (η^2^g) for the full RM-ANOVA are reported in the Results text. * For VAS pain, Week 3 was the earliest available assessment and was therefore used as the initial assessment; a preoperative VAS baseline was not available. Statistical significance was set at *p* < 0.05.

**Table 3:** Descriptive stratification of outcomes by fracture site (*N* = 30 patients).

Outcome	Femur (*n* = 20)	Tibia (*n* = 10)
Binary outcomes		
Radiographic union, *n* (%)	19/20 (95.0%)	8/10 (80.0%)
Short-term infection control, *n* (%)	19/20 (95.0%)	10/10 (100.0%)
Return to work or usual activity, *n* (%)	19/20 (95.0%)	8/10 (80.0%)
ASAMI functional—Excellent, *n*	15	5
ASAMI functional—Good, *n*	4	3
ASAMI functional—Poor, *n*	1	2
Continuous outcomes at Month 6, Mean ± SD		
ESR (mm/h)	22.6 ± 9.8	27.9 ± 14.4
CRP (mg/L)	2.7 ± 1.2	5.0 ± 5.5
RUST score	10.7 ± 1.0	10.0 ± 1.8
VAS pain score	0.5 ± 0.8	1.2 ± 1.5
EQ-5D-5L	0.87 ± 0.16	0.78 ± 0.27
Time to union (weeks)	18.1 ± 2.2	19.4 ± 3.5

Note: No formal between-group tests were conducted. Data are presented for descriptive purposes only. Baseline ESR: Femur 66.1 ± 9.9 mm/h, Tibia 71.2 ± 9.0 mm/h. Baseline CRP: Femur 36.6 ± 10.1 mg/L, Tibia 44.6 ± 12.1 mg/L. Baseline EQ-5D-5L: Femur 0.42 ± 0.11, Tibia 0.37 ± 0.16.

**Table 4:** Descriptive stratification of outcomes by pathogen resistance profiles (*N* = 30).

Outcome	Susceptible (*n* = 14)	MDR (*n* = 12)	XDR (*n* = 4)
Binary outcomes			
Radiographic union, *n* (%)	14/14 (100.0%)	9/12 (75.0%)	4/4 (100.0%)
Short-term infection control, *n* (%)	14/14 (100.0%)	11/12 (91.7%)	4/4 (100.0%)
Return to work or usual activity, *n* (%)	14/14 (100.0%)	9/12 (75.0%)	4/4 (100.0%)
ASAMI functional—Excellent, *n*	12	6	2
ASAMI functional—Good, *n*	2	3	2
ASAMI functional—Poor, *n*	0	3	0
Continuous outcomes at Month 6, Mean ± SD			
ESR (mm/h)	19.4 ± 3.9	30.4 ± 16.1	23.8 ± 5.4
CRP (mg/L)	3.1 ± 1.1	4.1 ± 5.2	3.0 ± 1.8
RUST score	10.3 ± 0.5	10.2 ± 2.0	11.5 ± 0.6
VAS pain score	0.4 ± 0.5	1.2 ± 1.5	0.5 ± 0.6
EQ-5D-5L	0.89 ± 0.08	0.76 ± 0.29	0.89 ± 0.16
Time to union (weeks)	17.3 ± 1.0	19.9 ± 3.6	19.0 ± 1.2

Note: No formal between-group tests were conducted. Data are presented for descriptive purposes only. XDR (*n* = 4) was too small for any meaningful comparison and is shown for completeness only. All three cases of nonunion, the single infection failure, and all three Poor ASAMI functional outcomes occurred in the MDR group. Baseline ESR: Susceptible 65.9 ± 7.2, MDR 72.4 ± 10.9, XDR 60.5 ± 9.1 mm/h. Baseline CRP: Susceptible 39.3 ± 12.9, MDR 40.4 ± 11.2, XDR 35.8 ± 5.0 mg/L. Baseline EQ-5D-5L: Susceptible 0.43 ± 0.11, MDR 0.37 ± 0.14, XDR 0.43 ± 0.17.

## Data Availability

The datasets generated and/or analyzed during the current study are available in the Figshare repository at: https://doi.org/10.6084/m9.figshare.31679785.

## Supplementary Materials

The following supporting information can be downloaded at https://www.mdpi.com/article/10.3390/healthcare14081091/s1: Supplementary Table S1. The completed STROBE checklist. Supplementary Figure S1. Custom threaded antibiotic-coated locking nail (TACLN). (A) Threaded inner rod with proximal and distal nuts prior to cement application. (B) Fabrication components: threaded rod, bone cement, gentamicin, and vancomycin. (C) Intraoperative PMMA mixing with antibiotic loading. (D) Cement mantle application using the chest tube mold on the sterile field. (E) Complete nail after coating.

## Abbreviations

The following abbreviations are used in this manuscript:

ACCIN	Antibiotic Cement-Coated Intramedullary Nail
AP	Anteroposterior
ASAMI	Association for the Study and Application of the Method of Ilizarov
BMI	Body Mass Index
CCI	Charlson Comorbidity Index
CI	Confidence Interval
CRP	C-Reactive Protein
EQ-5D-5L	EuroQol 5-Dimension
ESR	Erythrocyte Sedimentation Rate
IQR	Interquartile Range
LMIC	Low- and Middle-Income Country
MDR	Multidrug-Resistant
MIC	Minimum Inhibitory Concentration
MRSA	Methicillin-Resistant Staphylococcus aureus
MSSA	Methicillin-Susceptible Staphylococcus aureus
PMMA	Polymethyl Methacrylate
RUST	Radiographic Union Scale For Tibial Fractures
SD	Standard Deviation
STROBE	Strengthening the Reporting of Observational Studies in Epidemiology
TACLN	Threaded Antibiotic-Coated Locking Nail
VAS	Visual Analogue Scale
XDR	Extensively Drug-Resistant
