# Supplementary material for: Threaded Antibiotic-Coated Locking Nails in Osteomyelitis-Associated Long-Bone Non-Union: Short-Term Outcomes of a Prospective Cohort
Source: Healthcare (Basel). 2026 Apr 20;14(8):1091. doi: 10.3390/healthcare14081091 (PMC13115833; doi:10.3390/healthcare14081091)

Threaded Antibiotic-Coated Locking Nails in Osteomyelitis-Associated Long-Bone Non-Union: Short-Term Outcomes of a Prospective Cohort

Supplementary Table S1- STROBE Checklist — Cohort Study

Item No.	Recommendation	Reported	Location in Manuscript
TITLE AND ABSTRACT			
1a	Indicate the study's design with a commonly used term in the title or abstract	Yes	Title: '...A Prospective Cohort'; Abstract: 'prospective single-center cohort study'
1b	Provide an informative and balanced summary of what was done and what was found	Yes	Abstract — Background, Methods, Results, Conclusions
INTRODUCTION			
2	Background/rationale: Explain the scientific background and rationale for the investigation	Yes	Section 1 (Introduction), paragraphs 1–4
3	Objectives: State specific objectives, including any prespecified hypotheses	Yes	Section 1, final paragraph; Abstract (Conclusions)
METHODS			
4	Study design: Present key elements of study design early in the paper	Yes	Section 2.1: prospective single-center cohort; no control group explicitly stated
5	Setting: Describe the setting, locations, and relevant dates, including periods of recruitment, exposure, follow-up, and data collection	Yes	Section 2.1: Mercy Teaching Hospital, Peshawar, Pakistan; August 2025–February 2026
6a	Participants: Give the eligibility criteria, and the sources and methods of selection of participants. Describe methods of follow-up	Yes	Section 2.2: inclusion/exclusion criteria; all 30 enrolled completed 6-month follow-up
6b	For matched studies, give matching criteria and number of exposed and unexposed	N/A	Not applicable — single-arm cohort, no matching
7	Variables: Clearly define all outcomes, exposures, predictors, potential confounders, and effect modifiers. Give diagnostic criteria if applicable	Yes	Sections 2.4, 2.5.1–2.5.3: primary outcome defined; all variables listed with diagnostic criteria

Item No.	Recommendation	Reported	Location in Manuscript
8	Data sources/measurement: For each variable of interest, give sources of data and details of methods of assessment	Yes	Sections 2.4, 2.5.2, 2.5.3: prospective proforma; radiograph assessment protocol; EQ-5D-5L, VAS, ASAMI instruments described
9	Bias: Describe any efforts to address potential sources of bias	Yes	Section 2.5.2: blinded radiograph assessment; Section 4.6: selection bias, missing covariates, single-center acknowledged as limitations
10	Study size: Explain how the study size was arrived at	Yes	Section 2.1: no formal sample size calculation; explicitly stated as exploratory/pilot study
11	Quantitative variables: Explain how quantitative variables were handled in the analyses	Yes	Section 2.6: mean ± SD for normal distribution; median (IQR) otherwise; Shapiro-Wilk for normality
12a	Statistical methods: Describe all statistical methods, including those used to control for confounding	Yes	Section 2.6: RM-ANOVA, Greenhouse-Geisser correction, Bonferroni post-hoc, paired t-test, McNemar's exact test, Cohen's dz, eta-squared
12b	Describe any methods used to examine subgroups and interactions	Yes	Section 3.5: exploratory descriptive stratification by fracture site and resistance profile; no formal testing performed
12c	Explain how missing data were addressed	Yes	Section 3 (Results opening): no missing data — all 30 patients had complete data at all prespecified timepoints
12d	Cohort study: explain how loss to follow-up was addressed	Yes	Figure 1 (STROBE flow diagram): lost to follow-up = 0; discontinued = 0
12e	Describe any sensitivity analyses	N/A	No sensitivity analyses performed; acknowledged as a limitation of the exploratory design
RESULTS			
13a	Participants: Report numbers of individuals at each stage of study	Yes	Figure 1 (STROBE flow diagram): 40 screened, 10 excluded (6 ineligible, 4 declined), 30 analyzed
13b	Give reasons for non-participation at each stage	Yes	Figure 1: not meeting eligibility criteria (n=6); declined participation (n=4)
13c	Consider use of a flow diagram	Yes	Figure 1: STROBE flow diagram included
14a	Descriptive data: Give characteristics of study participants and information on exposures and potential confounders	Yes	Table 1: demographics, bone defect size, prior treatment, comorbidities, pathogen profile, resistance profile

Item No.	Recommendation	Reported	Location in Manuscript
14b	Indicate number of participants with missing data for each variable of interest	Yes	Section 3 (opening): no missing data for planned analyses; Table 1 footnote: smoking, open-fracture classification, soft-tissue staging not recorded
14c	Cohort study: summarize follow-up time	Yes	Section 2.1: follow-up period August 2025–February 2026; Section 3: all 30 patients completed 6-month follow-up
15	Outcome data: Report numbers of outcome events or summary measures over time	Yes	Table 2: longitudinal data at Baseline, Week 3, Week 6, Month 6; Sections 3.2–3.4: binary outcomes with proportions and 95% CIs
16a	Main results: Give unadjusted estimates and their precision (95% CI). Make clear which confounders were adjusted for	Yes	Table 2: mean change with 95% CI and effect sizes; Sections 3.2–3.4: proportions with 95% CI. No adjustment for confounders — single-arm design; acknowledged in Section 4.6
16b	Report category boundaries when continuous variables were categorized	Yes	Section 2.5.2: RUST ≥10 threshold for union; ASAMI functional and bone criteria defined in Sections 2.5.2–2.5.3
16c	If relevant, consider translating estimates of relative risk into absolute risk for a meaningful time period	N/A	Single-arm cohort; no comparative risk estimates. Absolute proportions with 95% CI reported throughout
17	Other analyses: Report other analyses done, including subgroup and sensitivity analyses	Yes	Section 3.5: exploratory descriptive stratification by fracture site (Table 3) and resistance profile (Table 4); explicitly framed as hypothesis-generating
DISCUSSION			
18	Key results: Summarize key results with reference to study objectives	Yes	Section 4 (Discussion opening paragraph): all primary endpoints summarized with reference to study aim
19	Limitations: Discuss limitations, including potential bias or imprecision, and both direction and magnitude of bias	Yes	Section 4.6: single-center, small sample, 6-month follow-up, no control group, RUST off-label in femur, missing covariates (smoking, open-fracture grade, soft-tissue staging), clinical LLD measurement, no formal subgroup testing
20	Interpretation: Give a cautious overall interpretation considering objectives, limitations, and similar studies	Yes	Sections 4.1–4.5: comparative framing against Conway [24], Garabano [28], Sen [9], Wang [25]; single-arm design caveat applied throughout

Item No.	Recommendation	Reported	Location in Manuscript
21	Generalizability: Discuss the generalizability (external validity) of the study results	Yes	Section 4.6: single-center LMIC setting limits generalizability; relevance to similar resource-constrained MDR/XDR settings discussed in Section 4.5
OTHER INFORMATION			
22	Funding: Give the source of funding and the role of the funders for the present study	Yes	Declarations section: no external funding; APC covered by Alfaisal University

N/A = Not applicable. Partial = item addressed but with acknowledged limitations noted in the manuscript.

Supplementary Figure S1. Custom threaded antibiotic-coated locking nail (TACLN). (A) Threaded inner rod with proximal and distal nuts prior to cement application(B) Fabrication components: threaded rod, bone cement, gentamicin, and vancomycin (C) Intraoperative PMMA mixing with antibiotic loading. (D) Cement mantle application using the chest tube mold on the sterile field. (E) complete nail after coating.

A

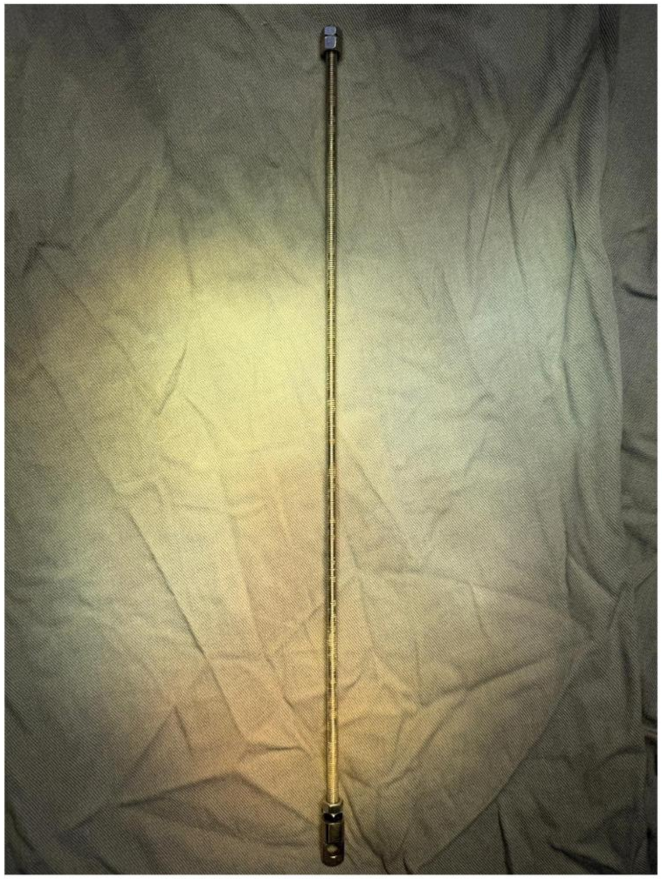

B

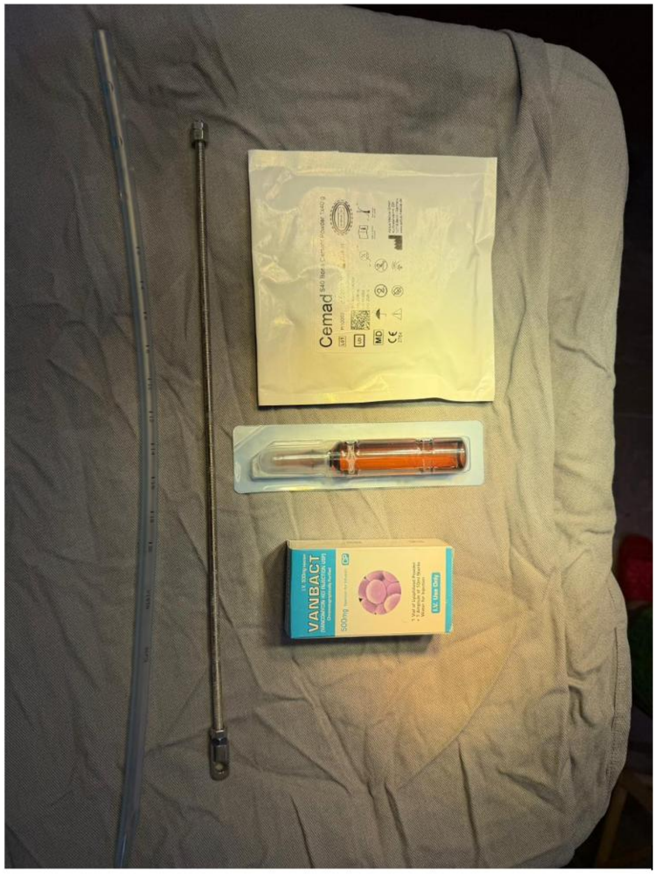

C

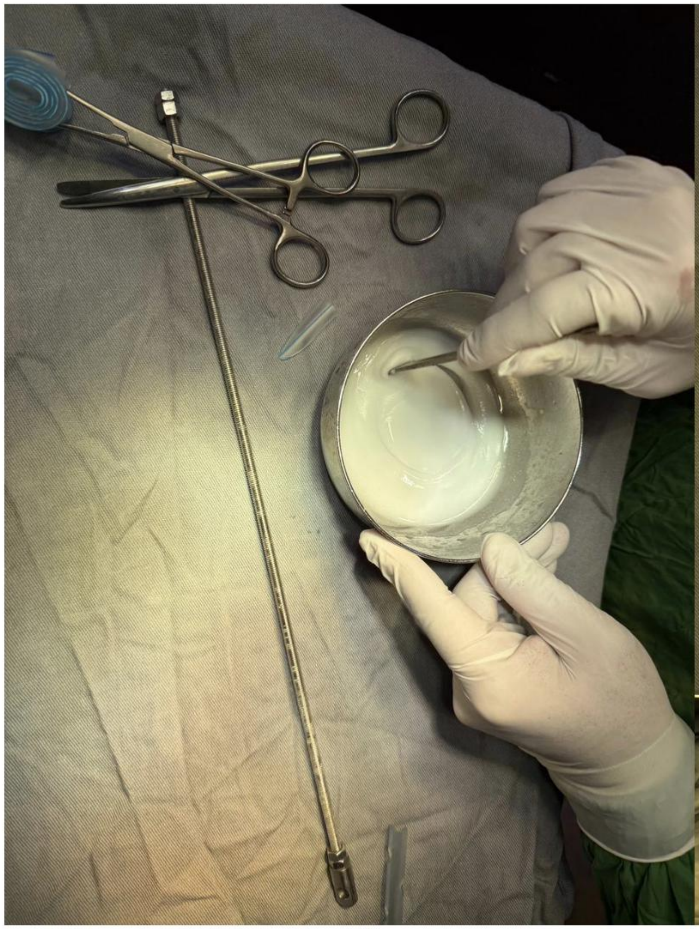

D

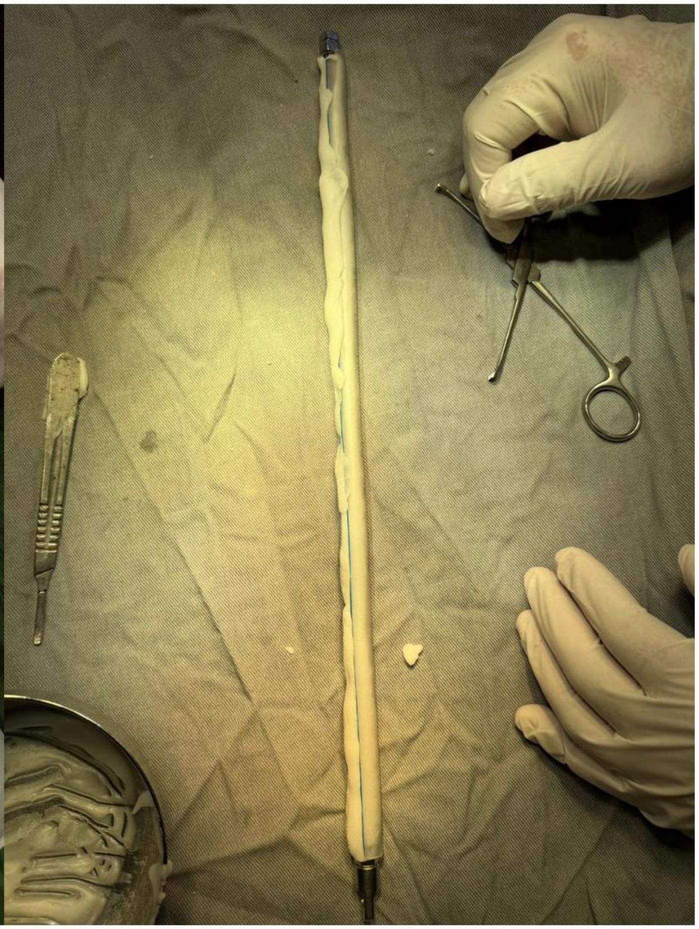

E

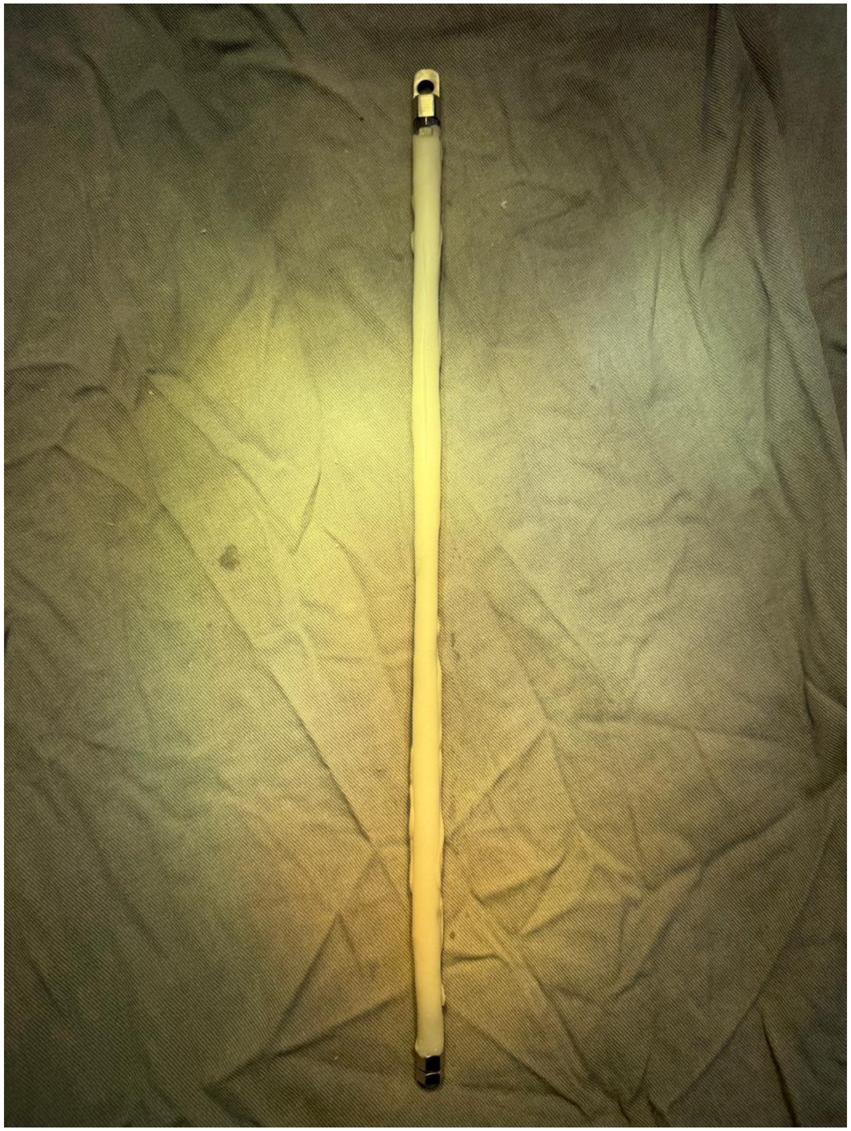

Supplement: Supplementary file 1 [file healthcare-14-01091-s001.zip › healthcare-4226562-supplementary.pdf]
